# Vibration and damping characteristics of 3D printed Kagome lattice with viscoelastic material filling

**DOI:** 10.1038/s41598-018-27963-4

**Published:** 2018-06-25

**Authors:** Rong Wang, Jianzhong Shang, Xin Li, Zirong Luo, Wei Wu

**Affiliations:** 1 College of Mechatronics and Automation, National University of Defense Technology, Changsha, Hunan 410073 China; 2 State Key Laboratory of Pulsed Power Laser Technology, Electronic Engineering Institute, Hefei, 230037 China

## Abstract

Constrained layer dampers (CLD) are in widespread use for passive vibration damping, in applications including aerospace structures. However, the introducing of the damping layer can reduce the stiffness of the sandwich structures. A viscoelastic material filling (VMF) is chosen to balance structural and vibrational performance of lattice truss in this work. The recently brought forward 3D Kagome truss with face sheet was manufactured by selective laser sintering technology and the thermosetting polyurethane was chosen as the viscoelastic filling material. A novel complex modal analysis finite element method for Hybrid composite lattice truss sandwich is introduced in this paper. Dynamic analysis experiment results show that the VMF method is found to be effective in reducing the vibration amplitude and it has the potential for band-gap design. The VMF method can provide high stiffness at low mass and considerable vibrational performance at low cost and it can be considered as a general vibration design method in lattice truss manufacture.

## Introduction

3D truss lattices have relatively high stiffness and yield strength that are achievable at low density. They play a significant role in achieving fuel efficiency goals^1–3^ and have been developed and optimized in various applications^4–6^. Unfortunately, lightweight 3D truss lattices structures are usually associated with relatively low damping^7–10^ which can cause early mechanical damage caused by resonant vibration^11^.

To seek optimal solutions satisfying both structural and vibrational requirements, an excellent combination of stiffness and damping can be obtained by choosing suitable materials and geometric configurations of the face sheets and cores.

The method of constrained layer damping (CLD) was introduced by Kerwin^12^ and it has been the most used technique in vibration suppression^13^. Many optimization techniques such as Genetic Algorithm^14^, Moving Asymptotes method^15^, Topology Optimization method^16^, modal strain energy method^17^ and recently Double Shear Lap-Joint-configuration^13,18^ were successfully adopted to optimize the location and dimensions of CLD, in order to maximize the structural damping while minimizing additional mass. However, the introducing of the damping layer can reduce the stiffness of the sandwich structures^11^.

In addition, filling of the core voids with foam or viscoelastic material have also been investigated in hybrid structures design to balance structural and vibrational performance of lattice truss. Zhang^19^ investigated that pyramidal lattice core sandwich panels filled with polyurethane foam have a greater load carrying capacity compared to the sum of the unfilled specimens and the filled polyurethane block. Li^20^ studied the frequency dependence of damping for foam-filled honeycomb sandwich beams by using the Ross–Ungar–Kerwin model. However, to the authors’ knowledge, there are only few reports on vibration damping optimization of composite sandwich structures order to improve the structural damping loss factors with honeycomb cores^13,18,20^, and much less for complicated lattice truss cores.

**Figure 1:**
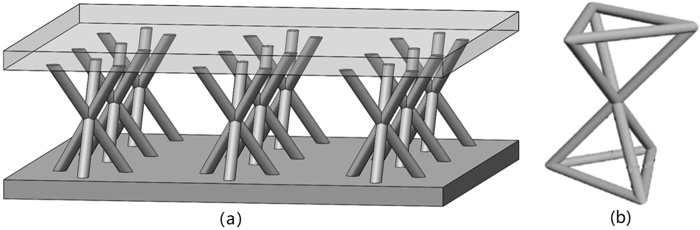
Kagome truss. (**a**) 3X3 Kagome lattice plate with face sheet, (**b**) single Kagome structure. Image acquisition tool: SolidWorks software (Dassault Systèmes SolidWorks Corp., USA).

The purpose of the present work is to explore a way to balance structural and vibrational performance of lattice truss by viscoelastic material filling (VMF). The VMF method is generally based on filling viscoelastic materials to the voids in the lattice structure so that the viscoelastic material can absorb the vibrational energy. Dynamic properties of this method is evaluated by the finite element method. The recently brought forward 3D Kagome^21,22^ truss (Fig. 1) with face sheet was taken as an example to demonstrate this new optimization method.

In this study we introduce two novel research strategies lacking in previous work: (1) this is the first work studied the mechanical properties of 3D printed complicated structures with viscoelastic material filling. Previous VMF work^20^ focused the honeycomb sandwich beams. A 3X3 3D Kagome structure with face sheet was manufactured and polyurethane is used as the viscoelastic solid for filling in this study. This design technique is theoretically analyzed by the finite element method and the constitutive model of the viscoelastic material is assumed as the standard linear solid from generalized Maxwell model^23^. (2) To introduce a new complex modal analysis method, Frequency domain dividing complex modal analysis method. The elastic modulus and damping ratio of viscoelastic solids cannot be directly taken into the matrix formula to solve eigenfrequency^24^, since they vary with frequency. This paper proposes an ideal of taking the elastic modulus and damping ratio of viscoelastic solids from each frequency segment into FEM model respectively, then use the window function to process the data, and get the final result of eigenfrequency.

In this paper we show for the first time that using the viscoelastic material filling in 3D truss lattices for vibration optimization other than using the damping layer. The viscoelastic material has good ability of vibration absorption. but it cannot be used independently in engineering for load resisting, because of its low elastic modulus^24^. The lattice structures have relatively high rigidity, so the VMF method can balance the structural and vibrational performance of 3D truss lattices.

To evaluate the effect of the design method, polyurethane is chosen as the viscoelastic filled material. The nylon PA6 is chosen as the material of 3D lattice structure. Kagome structure with face sheets were manufactured by the selective laser sintering technology and fixed vibration modal tests showed that compared with the traditional Kagome lattice plate, the acceleration amplitude of VMF Kagome lattice plate at natural frequency is reduced by 18.19 dB. The acceleration amplitude of VMF Kagome lattice plate at natural frequency is decreased by 6.03 dB, compared with solid plate.

This paper also find that the VMF method has the potential for band-gap design.

## Mechanical model of polyurethane viscoelastic solid

The viscoelastic material play an important role in vibration elimination and noise elimination. Thermosetting polyurethane viscoelastic solid have the advantages of: wide range of mechanical performance, strong adhesive ability, good aging resistance. So polyurethane is chosen as the viscoelastic filling material.

Viscoelastic materials have the characteristics of elastic modulus and loss factor varying with load frequency and environmental temperature, which has an important influence on the dynamic calculation of structures. The accuracy of measurement of elastic modulus and loss factor of viscoelastic material determines the accuracy of design evaluation.

In this paper, a simple method is used to obtain the frequency dependent characteristics of viscoelastic materials. The properties of viscoelastic materials are measured by stress relaxation test. The design does not involve the change of temperature, so the influence of temperature on the elastic modulus and the loss factor of the material is not considered.

Viscoelasticity is the property of materials that exhibit both viscous (ideal Newtonian liquid) and elastic (ideal elastic solid) characteristics when undergoing deformation. Viscoelastic substances behave as a combination of viscous and elastic. Some phenomena in viscoelastic materials are: (1) if the stress is held constant, the strain increases with time (creep); (2) if the strain is held constant, the stress decreases with time (relaxation); (3) the effective stiffness depends on the rate of application of the load; (4) if cyclic loading is applied, hysteresis (a phase lag) occurs, leading to a dissipation of mechanical energy. The study of viscoelasticity of solid matter usually assumes the following hypothesis: (a) continuity; (b) uniformity; (c) isotropic; (d) without initial stress and deformation.

The classic viscoelastic constitutive model consists of a spring element (elastic) and a dashpot (viscous). For a spring element, there are the following relations between stress and strain according to hook’s law:

1 $$\sigma =E\varepsilon $$

where *σ* is the stress, *ε* is strain, and E is Young’s modulus of elasticity. For a dashpot, the stress is proportional to the strain rate and can be regarded as:

2 $$\sigma =\eta \frac{d\varepsilon }{dt}=\eta \dot{\varepsilon }$$

In Equation 2, *η* is the coefficient of viscosity, and $\dot{\varepsilon }$ is the first derivative of strain to time, that is strain rate.

**Figure 2:**
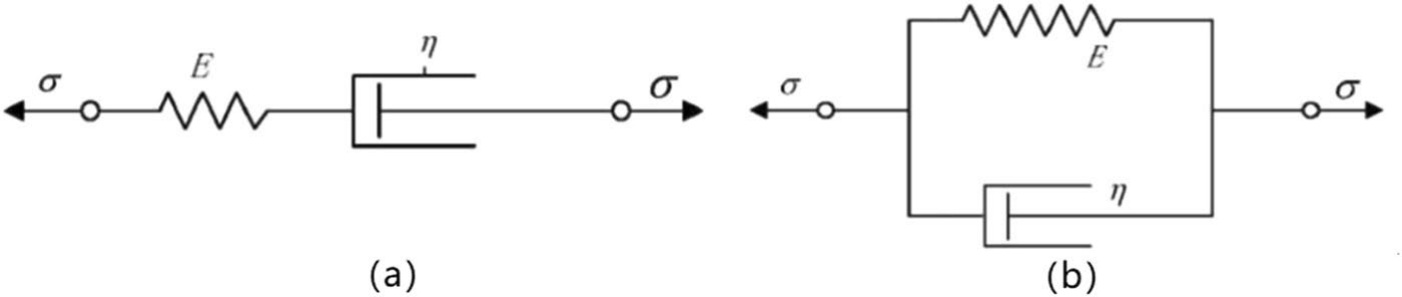
Maxwell (**a**) and Kelvin (**b**) model.

The properties of viscoelastic materials are some kind of combination of these two simple cases. The most commonly used two parameter models are Maxwell model and Kelvin model (Fig. 2). The constitutive equations of the two models can be regarded as:

Constitutive equations of Maxwell model:

3 $$\sigma +{p}_{1}\dot{\sigma }={q}_{1}\dot{\varepsilon }$$

Constitutive equations of Kelvin model:

4 $$\sigma ={q}_{0}\varepsilon +{q}_{1}\dot{\varepsilon }$$

The Maxwell model can reflect the relaxation phenomenon of the material, when loaded, as long as the existence of stress, the deformation will not stop, it is also known as the Maxwell fluid, which is obviously not in conformity with the preparation of polyurethane elastomer. The Kelvin model can eventually reach a limit when loading, thus reflecting the nature of the solid.

**Figure 3:**
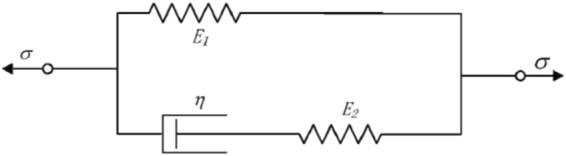
Standard 3-parameter viscoelastic solid model.

Xue^25^ studied that there is large deviations when representing viscoelastic properties with 2-parameter model while the 3-parameter model can describe the viscoelastic behavior of materials better. Figure 3 shows the standard 3-parameter viscoelastic solid model:

Its constitutive relation is:

5 $$\sigma +{p}_{1}\dot{\sigma }={q}_{0}\varepsilon +{q}_{1}\dot{\varepsilon }$$

where: $${p}_{1}=\frac{{\eta }_{1}}{{E}_{1}+{E}_{2}}$$; $${q}_{0}=\frac{{E}_{1}{E}_{2}}{{E}_{1}+{E}_{2}}$$; $${q}_{1}=\frac{{E}_{2}{\eta }_{1}}{{E}_{1}+{E}_{2}}$$

The stress relaxation properties are studied by using the 3-parameter viscoelastic solid model. When stress relaxation tests are applied, each particular strain value is applied. Corresponding to each particular strain value $\varepsilon (t)={\varepsilon }_{0}{\rm{H}}({\rm{t}})$, H(t) is the unit Heaviside step function defined as zero for t less than zero, one for t greater than zero, and 1/2 for t = 0^26^.

put $\varepsilon (t)={\varepsilon }_{0}{\rm{H}}({\rm{t}})$ into Equation 5 and use the Laplace transform we can get:

6 $$\sigma (1+{p}_{1}s)=({q}_{0}+{q}_{1}s){\varepsilon }_{0}/s$$

Inverse transformation and arranging:

7 $$\sigma (t)=\frac{{E}_{1}{E}_{2}}{{E}_{1}+{E}_{2}}{\varepsilon }_{0}+\frac{{E}_{2}^{2}{\varepsilon }_{0}}{{E}_{1}+{E}_{2}}{e}^{-t({E}_{1}+{E}_{2})/{\eta }_{1}}$$

Assume that: $$A=\frac{{E}_{1}{E}_{2}}{{E}_{1}+{E}_{2}}{\varepsilon }_{0}$$; $$B=\frac{{E}_{2}^{2}{\varepsilon }_{0}}{{E}_{1}+{E}_{2}}$$; $${p}_{1}={\eta }_{1}/({E}_{1}+{E}_{2})$$

Then:

8 $$\sigma (t)=A+B{e}^{-t/{p}_{1}}$$

Equation 8 is the material’s constitutive relation of stress relaxation in time domain. Where p_1_ represents the relaxation time of the material, and *ε*_0_ is the initial strain. Stress relaxation tests are required when determining the internal parameters.

The stress relaxation test is similar to the compression test. Three specimens of the same size (16 mm*16 mm*16 mm) were used in the test. Initially, compression test is performed on one of the specimens, with the compression speed of 1.4 mm/min. When the strain reaches a certain value, the strain remains constant (that is, the stamping head stays still), Observe and record the change of stress. At this point, the stress decreases as time goes on, since the relaxation occurs. When the total time of the experiment reaches a certain time (720 s), remove the load and change another specimen. Then continue to load at the original speed until the strain reaches second the set points and do the relax experiment again. Repeat the above process and measure the rest of the sample.

**Figure 4:**
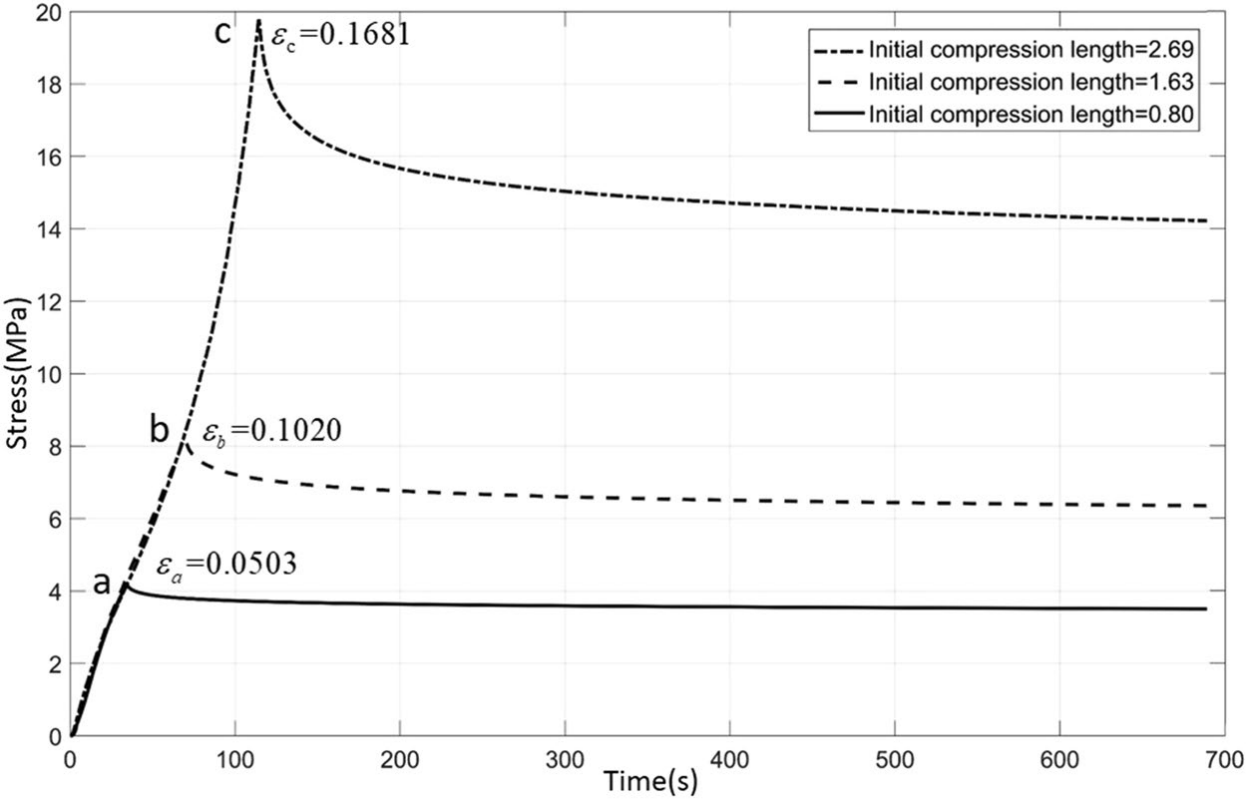
Compression and stress relaxation curves of polyurethane viscoelastic solid. Straight line, dotted line, dash dot are respectively corresponding to the compression and stress relaxation curves of the 1, 2, 3 specimens. Points a/b/c are the relaxation starting points for each tests.

Figure 4 illustrates the polyurethane viscoelastic solid strain relaxation curve. Three specimens were compressed to about 5%; 10%, 17% (corresponding to a/b/c points), then the stamping head is stopped. The left side of these curves from a/b/c points can be regarded as a material compression experiment while the right side are the stress relaxation curves. The stress relaxation curve of the material can be described by Equation 8. Fit the three stress relaxation curves by MATLAB respectively. Three fitting equations can be obtained. The corresponding fitting parameters are listed in Table 1. Nonlinear properties in polyurethane are ignored in this tests^25^.

**Table 1:** Curve fitting equation and parameters.

Number	Fitting equation	R-squre	A	B	P_t_
1	$\sigma (t)=14.194+5.412{e}^{-0.04387t}$	0.9176	14.194	5.412	22.795
2	$\sigma (t)=6.305+1.838{e}^{-0.03397t}$	0.9098	6.305	1.838	29.438
3	$\sigma (t)=3.491+0.651{e}^{-0.03562t}$	0.8932	3.491	0.651	28.074
**Number**	**Initial compression length**	**Initial strain**	**E**_**1**_ **(MPa)**	**E**_**2**_ **(MPa)**	**η(MPa · s)**
1	2.6896	16.81%	305.87	116.63	9630.72
2	1.6320	10.20%	273.86	79.83	10411.83
3	0.8048	5.03%	443.37	82.68	14768.38

Where R-square stands for the coefficient of determination is defined as the ratio of SSR to SST:

9 $$R\mbox{--}square=\frac{SSR}{SST}=1-\frac{SSE}{SST}$$

SSR is the sum of squares due to regression and SSE is the sum of squares due to error means: the square sum of the error of the corresponding points of the fitted data and the original data:

10 $$SSE=\sum _{i=1}^{n}{w}_{i}({y}_{i}-{\hat{y}}_{i})$$

SST, total sum of squares, is the sum of squares of the difference between the original data and the mean:

11 $$SST=\sum _{i=1}^{n}{w}_{i}({y}_{i}-\bar{y})$$

The coefficient of determination is a representation of the quality of curve fitting. From the Equation 11, we can know that the normal value range of the coefficient of determination is [0, 1]. The better the formula fits the data in experiment, the closer the value of R-square is to 1.

Take the average value of E1 E2 and η. Then constitutive equation of standard 3-parameter viscoelastic solid model is obtained:

12 $$\sigma +26.73\dot{\sigma }=73.10\varepsilon +9116.36\dot{\varepsilon }$$

## Design of 3D Kagome structure and viscoelastic material filling

2D Kagome structure originated as a traditional bamboo basket weave pattern and was identified by topology optimization as an optimal structure based on its elastic modulus for a range of fraction volumes^27^. The 3D variant was proposed by a recent research investigating^28^.

Compared with tetrahedral and pyramidal truss cores, 3D-Kagome truss core possesses the higher strength, greater resistance to plastic buckling and shows the excellent isotropic performance. This structure resembles the rod-like internal structure of cancellous bone and has been shown to exhibit exceptional strength properties in compression and shear^29^.

**Figure 5:**
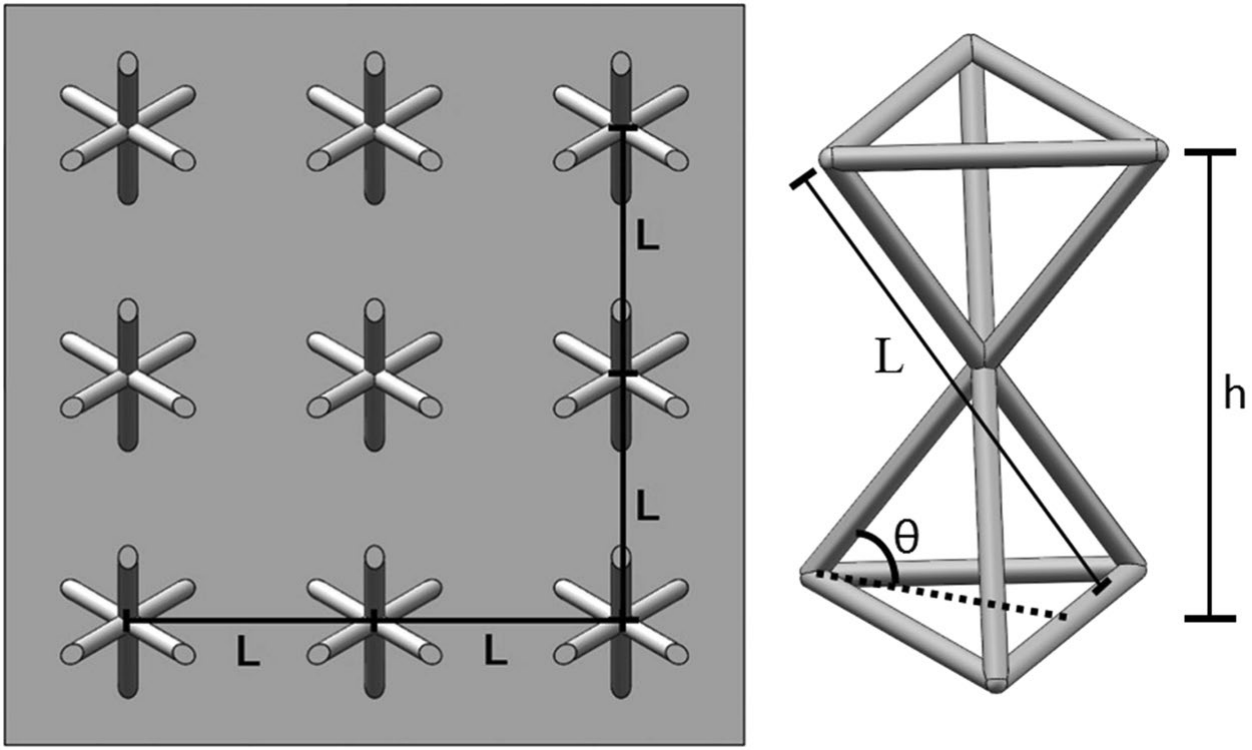
Parameters of Kagome truss. Image acquisition tool: SolidWorks software (Dassault Systèmes SolidWorks Corp., USA).

Figure 5 shows the 3D Kagome truss core and its main parameters. The 3D Kagome structure is formed by having pairs of tetrahedrons vertically inverted and rotationally offset from each other by 60°. A Kagome truss may be geometrically parameterized by internal angle of the truss structure θ, length of truss members l, cell height h and truss diameter d. In this study, the height h of the cell is fixed to a height of 11.5 mm, which is a typical core height in sandwich panels^29^. The diameter is 1.2 mm and internal angle of the truss structure θ is 60°. These two parameters play an important role in characterizing the mechanical properties and are considered key parameters in optimization studies. The value of d and θ are determined considering the previous work^30^. The length l of the truss members is a dependent variable and will be controlled only by the internal angle of the truss and cell height. In order to determine the mechanical properties of the single cell, it is built in its truss-based form with attached face sheets of 2 mm thickness^31^.

The 3D printing system chosen is a selective laser sintering system produced by FarsoonTech while the material selected is nylon. Its properties are listed in Table 2.

**Table 2:** Properties of Nylon PA6.

Thermal deformation temperature(0.46 MPa)	145 °C
Tensile Modulus	1646 MPa
Density	1002 Kg/m³
Poisson ratio	0.34

The selected viscoelastic filling material is thermosetting polyurethane. It is important to note that, when the thermosetting viscoelastic material is used for filling, the curing temperature should be less than 145 degrees Celsius, since the used nylon material has a thermal deformation temperature of 145 degrees Celsius. The properties of polyurethane are listed in Table 3.

**Table 3:** Properties of polyurethane in this paper.

Curing Temperature	120 °C
Constitutive equation	$\sigma +26.73\dot{\sigma }=73.10\varepsilon +9116.36\dot{\varepsilon }$
Density	1068 Kg/m³
Poisson ratio	0.475

**Figure 6:**
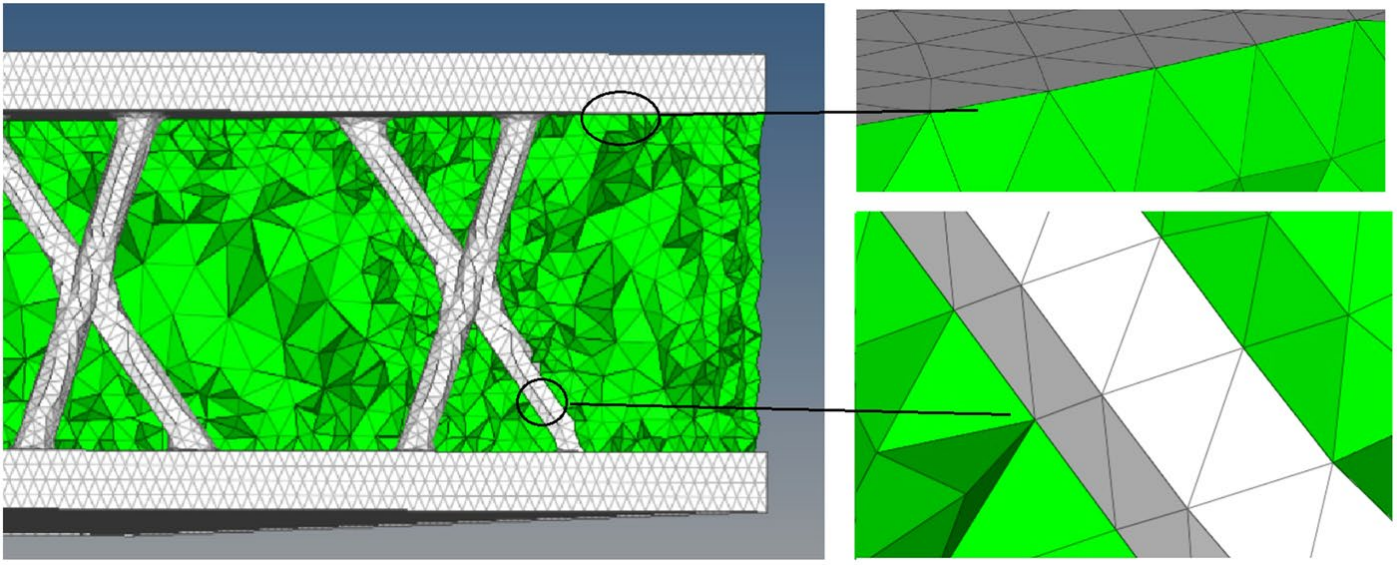
Combine surfaces with node merging. The white part stands for the 3D printed Kagome lattice with Nylon PA6 and the green part is the polyurethane solid. Image acquisition tool: HyperMesh (version 14.0, Altair, Troy, MI, USA).

We fill the polyurethane in the voids of the printed lattice structure after its printing. Figure 6 shows the finite element model of the hybrid structures in this study. It is important to simplify the structure and the connection between the lattice structure and the polyurethane because there are many complex structures in the model and the bonding surfaces between the 3D truss lattices and polyurethane are complicated. The case discussed in this paper is complete bonding without considering slip state, so the bonding surface of polyurethane and nylon is treated by way of node combination (Fig. 6).

## Finite element calculation and modal experiment

Modal analysis can be regarded as a coordinate transformation process, which is the decoupling process of the system vibration differential equation from the physical space through the modal transformation equation to the modal space. Since the modal transformation belongs to the linear transformation, the response of the system under external excitation can be considered as a linear superposition of its various modes, and the magnitude of the system depends mainly on the participation coefficients of the various modes. In general, the participation coefficients of the low order modes are much higher than those of higher order modes, and the dynamic response of the system can be obtained by only taking the superposition of the previous n order modes. The differential equation of vibration of a viscous damped vibration system with n degrees of freedom is^24^:

13 $$[m]\{\ddot{u}\}+[c]\{\dot{u}\}+[k]\{u\}=\{f(t)\}$$

$\{u\};\{\dot{u}\};\{\ddot{u}\}$ are the displacement matrix, velocity matrix and acceleration matrix described by physical coordinates respectively.

[m]; [c]; [k] represent the mass matrix, damping matrix and stiffness matrix of the system, respectively.

{f(t)} is the external excitation matrix.

For free vibration:

14 $$[m]\{\ddot{u}\}+[c]\{\dot{u}\}+[k]\{u\}=\{0\}$$

Assume

15 $$\{u\}=\{U\}{e}^{st}$$

where: U is a time independent vibration mode function; s is complex number

Substituting Equation 15 into Equation 13

16 $$([m]{s}^{2}+[c]s+[k])\{u\}=\{f(t)\}$$

The eigenequation is

17 $$[m]{s}^{2}+[c]s+[k]=\{0\}$$

The Equation 13 is transformed by Laplace:

18 $$([m]{s}^{2}+[c]s+[k])\{u(s)\}=\{f(s)\}$$

where: s is the Laplace coefficient

19 $$\{f(s)\}=L[\{f(t)\}]$$

Through this transformation, the problem of solving differential Equations (13) is transformed into the problem of solving algebraic Equation 18. For free vibration:

20 $$[m]{s}^{2}+[c]s+[k]=\{0\}$$

This is the characteristic Equation 17. Then, the eigenvalue problem of structural dynamical systems is the root of the homogeneous algebraic equations for Laplace differential equations. For single degree of freedom systems, these two roots are:

21 $${s}_{1,2}=-\,\frac{c}{2\,m}\pm \frac{\sqrt{{c}^{2}-4\,km}}{2\,m}=-{\omega }_{0}\pm j\,{\omega }_{0}\sqrt{1-{\xi }^{2}}$$

where: $${{\omega }_{0}}^{2}=\frac{k}{m}$$ $$\xi =\frac{c}{{c}_{0}}=\frac{c}{2\sqrt{km}}=\frac{c}{2m{\omega }_{0}}$$

S_1_ and S_2_ are conjugate complex numbers, and the real part is the attenuation factor, which reflects the damping of the system, and the imaginary part represents the natural frequency of the damped system^32^.

Viscoelastic materials exhibit behavior somewhere in between that of purely viscous and purely elastic materials, exhibiting some phase lag in strain. Complex modulus is used in modal analysis:

22 $${G}^{\ast }(i\omega )={G}_{1}(\omega )+i{G}_{2}(\omega )$$

G^*^ (i*ω*) is the complex modulus. *G*_1_ (*ω*) is the real part, reflecting the same phase change relation between stress and strain. *G*_1_ (*ω*) is also known as the storage modulus, which is applied to the calculation of elastic modulus; *G*_2_ (*ω*) is the imaginary part and the phase difference between stress and strain is PI/2. *G*_2_ (*ω*) is also called loss modulus

23 $$\eta (\omega )={G}_{2}(\omega )/{G}_{1}(\omega )$$

η is called damping factor.

For Standard 3-parameters viscoelastic solid model:

24 $${G}_{1}(\omega )={p}_{1}{q}_{1}{\omega }^{2}/(1+{{p}_{1}}^{2}{\omega }^{2})$$

25 $${G}_{2}(\omega )={q}_{1}\omega /(1+{{p}_{1}}^{2}{\omega }^{2})$$

**Figure 7:**
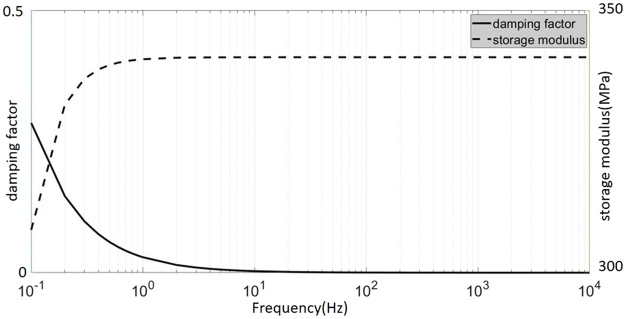
Storage modulus and loss factor vary with frequency. Straight line stands for damping factor. Dotted line stands for storage modulus.

The common finite element modal calculation generally assumes that the modulus of elasticity of the material is constant. This assumption does not hold true in the presence of viscoelastic materials. As shown in Fig. 7, the elasticity modulus and damping factor of the viscoelastic material involved in the paper varies considerably within the frequency band (0~10000 Hz). The changing of viscoelastic material’s elasticity modulus and damping factor should be considered when designing the viscoelastic material filling 3D truss lattices.

**Figure 8:**
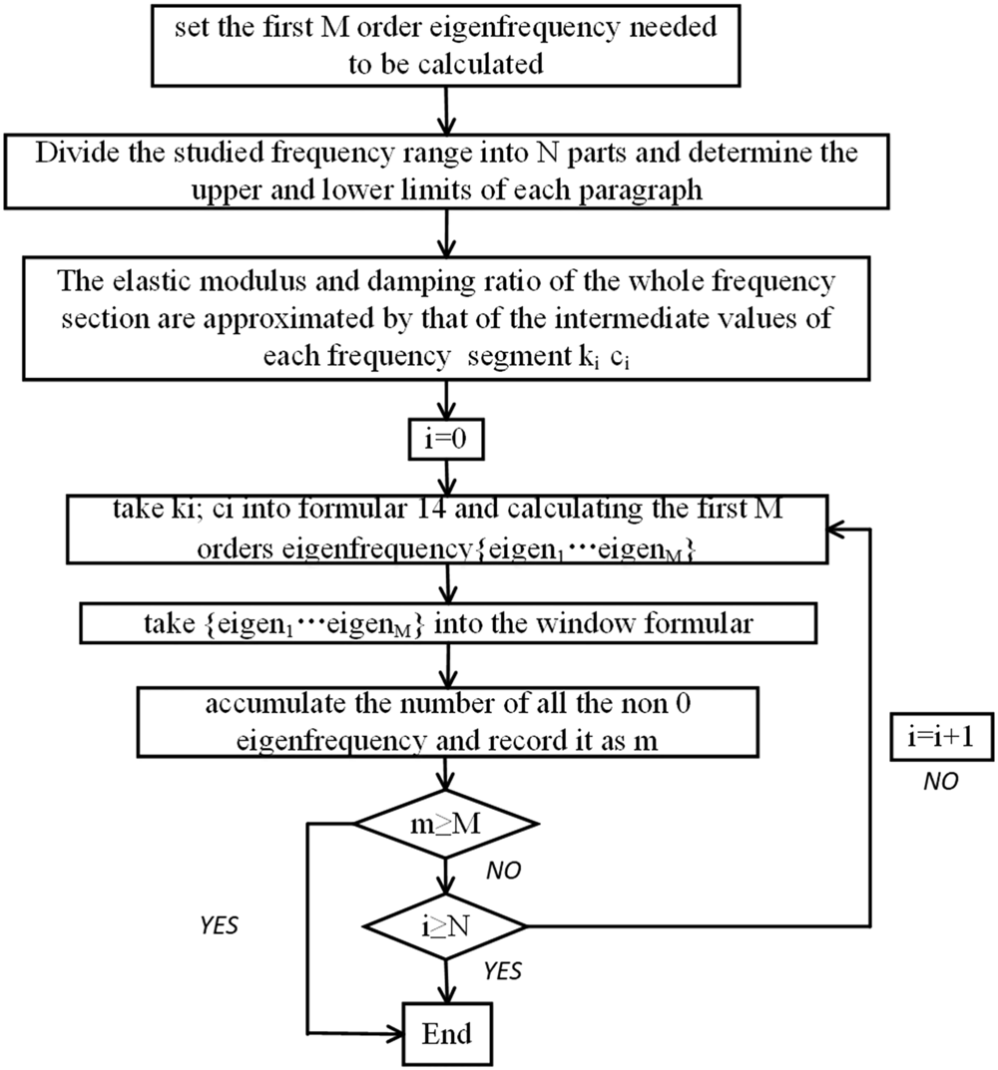
Flow chart of the complex modal calculation.

Figure 8 shows the flow chart of the complex modal calculation. Compared with other complex modal calculation methods^24^, this simple method can effectively control the amount of calculation and greatly shorten the computation time. Eigenfrequency calculation can be time consuming (there are 0.6 million elements in this finite element model). In such a large calculation, time saving is very important.

Suppose the first M orders Eigen frequency needs to be calculated and the frequency of interest is classified as N, and the frequency of each corresponds to [freqLi, freqUi),

Eigenfrequency window selection function

26 $$W(eige{n}_{ij})=\{\begin{array}{c}eige{n}_{ij},\,freq{L}_{i}\le eige{n}_{ij} < freq{U}_{i}\\ 0,\,else\end{array}$$

In this paper, first 3 orders of modes of VMF Kagome plate are calculated. Firstly, we set M = 3 and N = 5. We divide the interested frequency domain (0.1, 10000) into five sections, that is, (0.1, 1), [1, 10), [10, 100), [100, 1000), [1000, 10000). The median of each frequency band is introduced into Equations 23 and 24 for the calculation of approximate damping factor and storage for each frequency section. Take the results of the calculation to c and k in Equation 20. Then start the loop in Fig. 8. In fact, we find out that the eigenfrequency calculation results of first four sections are not in the corresponding frequency domain, so after the window function is processed, they turns into 0, and only the fifth frequency domain is really recorded in the result of the calculation.

The method of frequency segmentation is the average allocation of frequency segments on logarithmic function. Other frequency segmentation methods can be applied like segmentation according to the mechanical properties of materials or the detailed segmentation at a special frequency section.

This is the first work studied the mechanical properties of 3D printed complicated structures with viscoelastic material filling. Previous method can be very time consuming in the complicated finite element model because of the excessive number of iterations^24^. So a complex modal analysis method with seldom and predictable iterations is needed in this kind of research. The upper limit of the number of iterations in this article is 5.

The vibration modal analysis equipment used in this paper is DEWETRON dynamic signal analyzer. It mainly includes the hammer with force sensor, acceleration sensor, integrated charge amplifier, dynamic signal analyzer and modal analysis software. The type of hammer is Dytran 5800B4, and the sensitivity is 42.35 mV/N. The sensor is a piezoelectric accelerometer, Dytran 3035B, and the sensitivity is 92.85 mV/g. Hitch the specimen to the steel frame with the cord fastened and a small circular magnet (0.75 g) is glued on the test piece with epoxy resin.

After setting the relevant parameters and arranging the knocking point, the hammer is used to hit the reference point take the average value after 10 times knocking and measuring.

The hammer force generates a pulse signal, then the pulse signal is transmitted to the charge amplifier through the force sensor. Meanwhile, the accelerometer collects vibration signal of the reference point through the acceleration sensor, then the vibration signal is transmitted to the charge amplifier. These two signals are amplified and transmitted directly to the dynamic signal analyzer for post processing analysis to obtain the frequency response function and the related modal parameters. The torsional mode of the structure cannot be measured in this experiment. In the experiment, the excitation point is the geometric is the projected point on one side of face sheet from center of the Kagome in the first row and the second column (shown in Fig. 5), and the measuring point is the projection of that geometric center on the other face sheet.

Table 4 shows the calculated values and experimental results of the first three order natural frequencies of two kinds of plates. The natural frequencies are calculated and arranged in the finite element software Hypermesh in accordance with the method of Fig. 8. It can be seen that the calculated values are in good agreement with the experimental ones, and the error is less than 8%.

**Table 4:** The calculated values and experimental results of the three order natural frequencies of two kinds of plates.

Mode	Kagome lattice plate			VMF Kagome lattice plate		
	Calculated value (Hz)	Experimental value (Hz)	Error	Calculated value (Hz)	Experimental value (Hz)	Error
1	1653.3	1658	0.28%	2463.0	2641	6.74%
2	2052.5	1907	7.63%	3451.1	3231	6.81%
3	2447.2	2633	7.06%	3671.9	3525	4.17%

Another thing to note is that the over 1000 Hz data of material properties of polyurethane are mainly used in the calculation process. When the frequency range of study is greater than 1000 Hz, the damping factor of the material is very small (η < 3*10–5). In fact, even assuming that the material had a large damping factor in very high frequency range and that such data is taken into software calculations, the final natural frequencies are less affected (assuming that the damping factor of the material is 0.5 at high frequency and that the free modal frequency is within 3% of the actual calculation error). Although the damping factor in the material has limited influence on the value of the natural frequency, it has a great influence on the amplitude of vibration.

**Figure 9:**
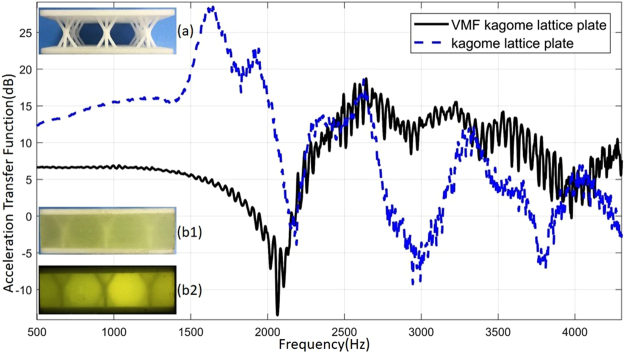
Acceleration transfer function for 2 types of plate in free modal tests. (**a**) Kagome lattice plate, (**b1**) VMF Kagome lattice plate under natural light, (**b2**) VMF Kagome lattice plate with light passing through. Straight black line stands for acceleration transfer function for VMF Kagome lattice plate. Dotted blue line stands for acceleration transfer function for kagome lattice plate.

Figure 9 is a comparison of acceleration transfer function of Kagome lattice plate and that of VMF Kagome lattice plate. The acceleration transfer function of Kagome lattice plate reached the peak value at 1658Hz, 1907Hz and 2633 Hz respectively, the corresponding amplitude were 28.77 dB, 22.78 dB, 18.60 dB. While the acceleration transfer function of VMF Kagome lattice plate reached the peak value at 2641 Hz, 3231 Hz, 3525 Hz respectively, the corresponding amplitude were 18.74 dB, 15.53 dB, 14.22 dB. At low frequencies (before 2100 Hz), the acceleration amplitudes of VMF Kagome lattice plate are significantly smaller than those of Kagome lattice plate, while at high frequencies (greater than 2100 Hz), the amplitudes of VMF Kagome lattice plate are generally greater than the original ones. Another thing to note is that, at 2050 Hz, the acceleration transfer function of the VMF Kagome lattice plate is only −14.4 dB, which means it has a noticeable shock absorption capability at that frequency.

Figure 9 illustrates the shock absorption capability of VMF Kagome lattice plate to a certain extent, but in fact, for mechanical systems, there is a transfer function:

27 $$[H(s)]=\frac{1}{[m]{s}^{2}+[c]s+[k]}$$

Adding viscoelastic material to the lattice element greatly influenced the mass matrix of lattice and plate. Wang^33^ carried out that the damping variation was found to be effective in reducing the amplitude without significantly shifting the natural frequency of the cantilever when he added extra mass balls to the honeycomb beams. To further illustrate the problem, another solid plate is made. The density of nylon (1002 kg/m^3^) used in this paper is similar to that of polyurethane density (1068 kg/m^3^). Therefore, the influence of mass matrix on transfer function can be neglected in the experimental comparison.

The natural frequencies of solid plates are much higher than those of Kagome plates (the first three natural frequencies are 4453 Hz, 5643 Hz and 8085 Hz), so we choose constrained modal analysis for comparison. The fixed way of constrained modal analysis is: put the lower edge (Fig. 5) in the fixture and the clamping depth is 8 mm. The measuring point and the exciting point remain unchanged.

**Figure 10:**
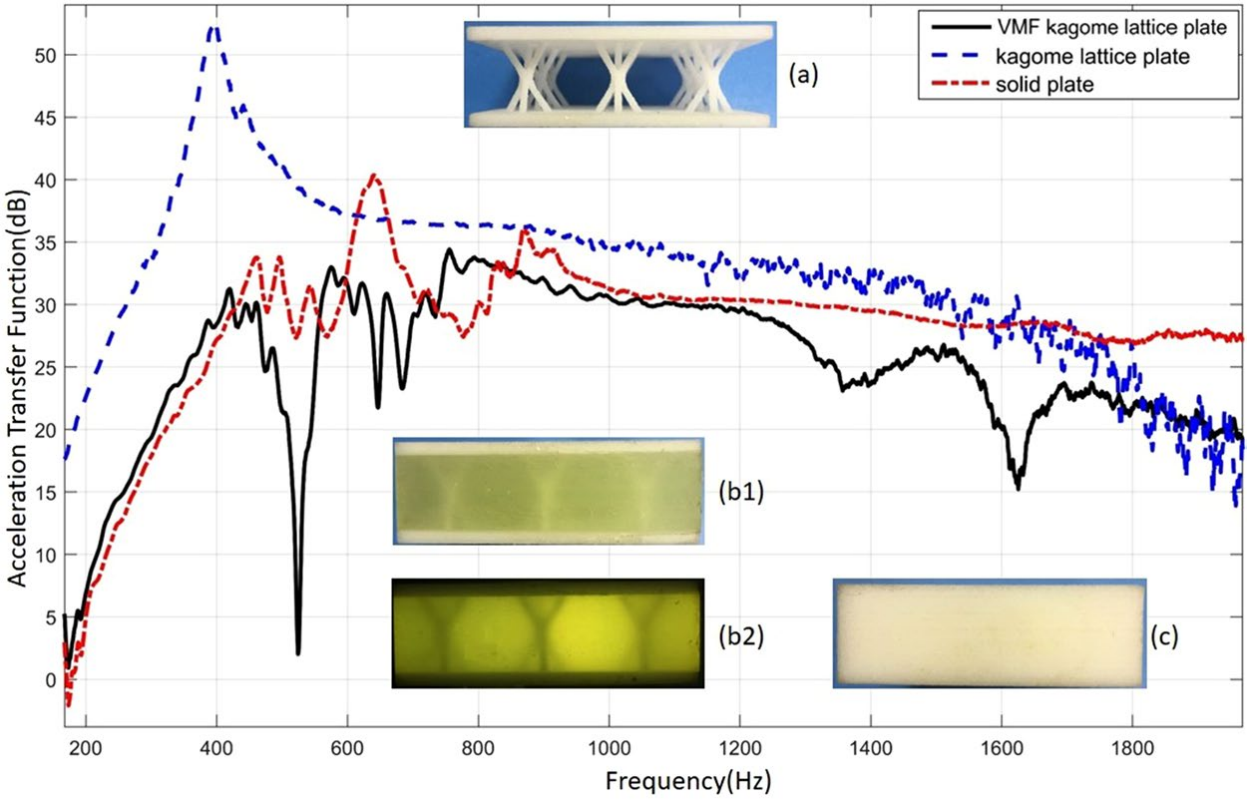
Acceleration transfer function for 3 types of plate under fixed support. (**a**) Kagome lattice plate, (**b1**) VMF Kagome lattice plate under natural light, (**b2**) VMF Kagome lattice plate with light passing through, (**c**) solid plate. Straight black line stands for acceleration transfer function for VMF kagome lattice plate. Dotted blue line stands for acceleration transfer function for Kagome lattice plate. Red dash dot stands for acceleration transfer function for solid plate.

Figure 10 shows acceleration transfer function for 3 types of plate under fixed support. In free modal experiment, the specimen was suspended in the air by a cord. When the hammer strikes the specimen, most of the energy is converted to the motion of the specimen. When the specimen is fixed, the specimen cannot move freely, so the energy is dissipated mainly by vibration. Therefore, the amplitude of the acceleration transfer function in the fixed mode experiment is much larger than that in the free mode. Due to the change of the fixed form and the prestress brought by the fixture, the frequency of maximum amplitude of the acceleration transfer function of the three plates is closer to that of the free mode. At most frequencies, the amplitude of VMF Kagome plate is much less than that of Kagome plate, there is only one resonance frequency at 397.3 Hz between 200–1800 Hz and the amplitude is as much as 52.51 dB. In contrast, the amplitude of the solid plate and VMF Kagome plate is much smaller, consistent with the previous research by Wang^33^, the addition of the mass can lead to the amplitude reduction in vibration. VMF Kagome plates and solid plate have almost the same mass matrix. Their different vibration characteristics are due to the mechanical properties of viscoelastic material. The maximum amplitude of VMF Kagome plate is 34.32 dB at 755.6 Hz, while the maximum amplitude of solid plate is 40.35 dB at 639.6 Hz. For this observation point, the amplitude at natural frequency is reduced by 6.03 dB. There is a distinct band-gap at 524.3 Hz.

## Discussion and Application

**Figure 11:**
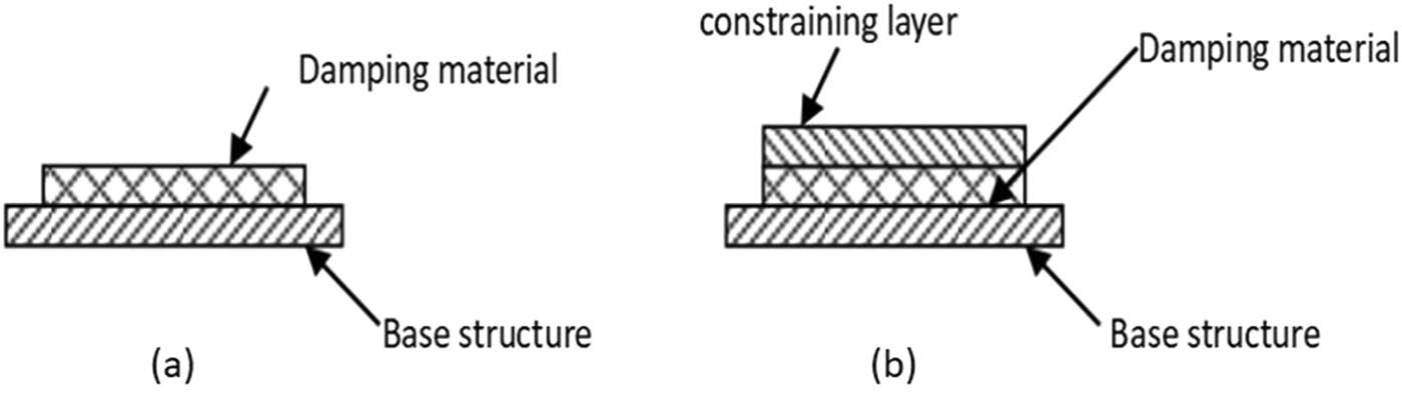
Free damping layer (**a**) and constrained damping layer (**b**).

Passive damping as a technology has been dominant in the non-commercial aerospace industry since the early 1960s^34^. Depending on the layout method, the damping layer can be divided into two types (Fig. 11). One is that the damping layer is attached to the surface of the main structural layer, as shown in Fig. 11(a). The damping layer can be deformed freely, so it is called the “free damping layer”. When the structure is excited, the damping layer mainly produces tensile compression deformation. Another kind of damping application is to paste a layer of material similar to the material of the main structure on the surface of the free damping layer, as shown in Fig. 11(b). When the structure is excited, the damping layer is fixed by two solid plate constraints, mainly produce shear deformation, so called “constrained damping layer”. In general, the “constrained damping layer” is better than the “free damping layer” in damping effect. VMF can be regarded as a special “constrained damping layer”, and the damping material receives more constraints and may cause more types of deformation. Compared with the traditional methods, the greatest advantage of the VMF method is that it can guarantee the component to have better rigidity. Since the lattice structure itself has very high specific stiffness, and it is a consensus that adding material to the existing structure cannot reduce its strength or stiffness^35^.

The complex modal calculation method introduced in this paper is not limited to the mechanical model obtained by stress relaxation test. The frequency dependent elastic moduli and damping ratios of materials obtained with other methods (e.g. viscoelastic spectrometers^36^) can also be calculated by this method.

In fact, compared with polyurethane in other literatures^25^, the damping of polyurethane material used in this paper is relatively small. Polyurethane viscoelastic solid have wide range of mechanical performance, so we can reasonably guess that by making more suitable polyurethanes (or other viscoelastic solids), the damping performance of VMF can be manifested more clearly.

In order to reduce the influence of mass distribution on damping effect, nylon, whose density is similar to polyurethane material density, is chosen as the printing material of lattice structure. In fact, this kind of viscoelastic material can also be used in metal printing parts, which can reflect both the weight reduction performance of 3D printing and the vibration optimization performance of VMF method.

**Figure 12:**
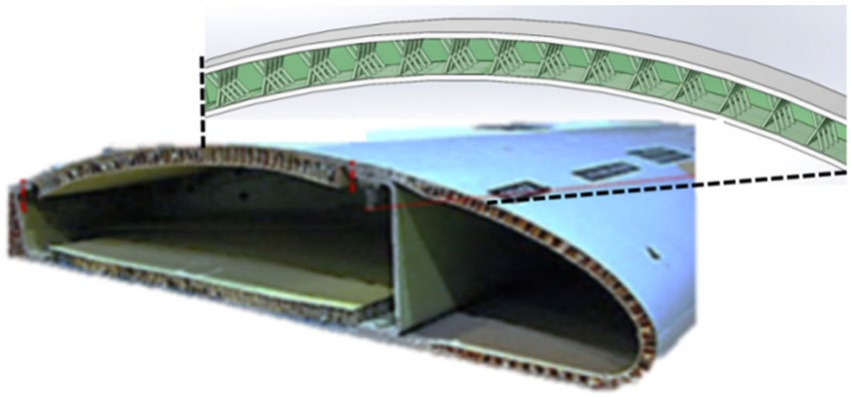
The proposed CAD model of wing with VMF Kagome structure. Image acquisition tool: SolidWorks software (Dassault Systèmes SolidWorks Corp., USA).

Additive manufacturing (AM) techniques enable to build complex lattice components at the micrometer length scale with high accuracy at acceptable costs. However, a reduction in mass may lead to new vibration problems. Filling the lattice with viscoelastic material can effectively improve the vibration characteristics of the parts. Based on the results from the experiments, a CAD model of the wing was established. The design was modified with a groove at the open end of the wing to allow the attachment of the wing unto the fuselage. The proposed CAD model is as shown in Fig. 12. The wing should be light and strong since the small Unmanned Aerial Vehicles (UAVs) has limited power. Meanwhile, effective reduction of unwanted vibrations is critical for stability control of aircraft. The VMF Kagome can provide large stiffness at low mass and reduce the vibration so it fits the UAVs perfectly. The VMF method can be very helpful in spacecraft design such as UVAs, satellites and rockets.

## Conclusions

In this study, modal test is carried out to investigate the vibration damping and Eigenfrequency of hybrid composite Kagome truss sandwich panels with viscoelastic material filling. Thermosetting polyurethane viscoelastic solid is chosen as the viscoelastic filling material. Material properties of polyurethane are obtained by the stress relaxation test. FEM model is established for theoretical analysis and the result of calculated eigenfrequency fit well with the experimental eigenfrequency value. Dynamic analysis experiment results show that the VMF method is found to be effective in reducing the amplitude without significantly shifting the natural frequency. Compared with the traditional Kagome lattice plate, the acceleration amplitude of VMF Kagome lattice plate at natural frequency is reduced by 18.19 dB, and the acceleration amplitude at natural frequency is decreased by 6.03 dB, compared with solid plate in fixed modal test.

This paper also find that the VMF method has the potential for band-gap design. It is found that the VMF method has the ability to obtain a band gap at a relatively low frequency in both the free and constrained modal analysis. However, the specific reasons for the frequency band gap and how can the designers get the band gap at desired frequency through choosing corresponding kind of viscoelastic material and lattice structure is still unknown. So additional work is currently undertaken to solve this problem.

The insertion of the viscoelastic material inside the face sheets and core of the sandwich panel increases the extra mass. Another work is currently undertaken to put viscoelastic material in 3D printed metal lattice to illustrate that the VMF method can play a better role in light weight design.

## Methods

### Materials

Commercially available nylon PA6 (FarsoonTech) was used in this study. The raw materials for preparation of polyurethane were Castor oil (molecular weight 933, average functional degree 27, equivalent molecular weight 345, analytically pure, crosslinking agent, Kemiou Chemical Reagent Co. China), polymer 8617 (MDI, molecular weight 1000, NCO content 23%), polymer 8618 (MDI, molecular weight 4000, content of NCO 17%), polymer 8608 (MDI, molecular weight 1000, NCO content 20%) produced by WanHua Chemical Co. China and DMTDA (curing agent, YaRui Chemical Co. China).

### 3D printer

Farsoon 403 P produced by FarsoonTech, China, was used for printing Kagome lattice.

### Dynamic tests

The vibration modal analysis equipment used in this paper was DEWETRON dynamic signal analyzer (DEWE2) produced by DEWETRON GmbH, Austria. Hammer with force sensor was Dytran 5800B4 produced by Dytran Instruments, Inc. USA and the sensitivity is 42.35 mV/N. Acceleration sensor was Dytran 3035B produced by Dytran Instruments, Inc. USA and the sensitivity is 92.85 mV/g.

## References

[1] Park J-H, Kim KJ (2013). Optimal design of camber link component for light weight automobile using CAE (Computer Aided Engineering). International Journal of Precision Engineering and Manufacturing.

[2] Dornfeld DA (2014). Moving towards green and sustainable manufacturing. International Journal of Precision Engineering and Manufacturing-Green Technology.

[3] Zhang Q (2015). Bioinspired engineering of honeycomb structure – Using nature to inspire human innovation. Progress in Materials Science.

[4] Wadley HN (2006). Multifunctional periodic cellular metals. Philos Trans A Math Phys Eng Sci.

[5] Queheillalt DT, Wadley HNG (2009). Titanium alloy lattice truss structures. Materials & Design.

[6] Deshpande NAF (2001). Collapse of truss core sandwich beams in 3-point bending. International Journal of Solids and Structures.

[7] Gibson RF (2010). A review of recent research on mechanics of multifunctional composite materials and structures. Composite Structures.

[8] Finegan IC (1999). R. F. G. Recent research on enhancement of damping in polymer composites. Composite Structures.

[9] Lakes RS (2002). High Damping Composite Materials: Effect of Structural Hierarchy. Journal of Composite Materials.

[10] Chandra R, Singh SP, Gupta K (1999). Damping studies in fiber-reinforced composites – a review. Composite Structures.

[11] Yang J-S (2016). Hybrid lightweight composite pyramidal truss sandwich panels with high damping and stiffness efficiency. Composite Structures.

[12] Kerwin EM (1959). Damping of Flexural Waves by a Constrained Viscoelastic Layer. The Journal of the Acoustical Society of America.

[13] Aumjaud P, Smith CW, Evans KE (2015). A novel viscoelastic damping treatment for honeycomb sandwich structures. Composite Structures.

[14] Boucher MA, Smith CW, Scarpa F, Rajasekaran R, Evans KE (2013). Effective topologies for vibration damping inserts in honeycomb structures. Composite Structures.

[15] Zheng Linga XR, Yi W, El-Sabbagh A (2011). Topology optimization of constrained layer damping on plates using Method of Moving Asymptote (MMA) approach. Shock and Vibration.

[16] Yi-Cheng Chen S-CH (2002). An optimal placement of CLD treatment for vibration suppression of plates. International Journal of Mechanical Sciences.

[17] Kienholz CDJADA (1982). Finite Element Prediction of Damping in Structures with Constrained Viscoelastic Layers. AIAA Journal.

[18] Madeira JFA, Araújo AL, Soares CMM, Soares CAM, Ferreira AJM (2015). Multiobjective design of viscoelastic laminated composite sandwich panels. Composites Part B: Engineering.

[19] Zhang G (2014). Energy absorption and low velocity impact response of polyurethane foam filled pyramidal lattice core sandwich panels. Composite Structures.

[20] Li Z, Crocker MJ (2006). Effects of thickness and delamination on the damping in honeycomb–foam sandwich beams. Journal of Sound and Vibration.

[21] Wang J, Evans AG, Dharmasena K, Wadley HNG (2003). On the performance of truss panels with Kagomé cores. International Journal of Solids and Structures.

[22] Hyun S, Karlsson AM, Torquato S, Evans AG (2003). Simulated properties of Kagomé and tetragonal truss core panels. International Journal of Solids and Structures.

[23] Gutierrez-Lemini, D. *Constitutive Equations in Differential Operator Form,1 Engineering Viscoelasticity*. *3*, *53–93* (Springer, 2014).

[24] Feng, T. *Analysis and optimization of viscoelastic damping structures in vibration and noise reduction*. Master thesis, Shanghai Jiao Tong University (2010).

[25] Chao, X. Q. *Mechanical properties study for sandwich plate system*. Doctor thesis, Harbin Engineering University (2013).

[26] Lakes, R. *Phenomena,1. Viscoelastic properties of materials. 1*, *1*–*12* (Cambridge University Press, 2009).

[27] Nathan Wicks JWH (2000). Optimal truss plates. International Journal of Solids and Structures.

[28] Hyun SaT (2002). S. Optimal and Manufacturable Two dimensional, Kagome-like Cellular Solids. Journal of Materials Research.

[29] Ullah I, Elambasseril J, Brandt M, Feih S (2014). Performance of bio-inspired Kagome truss core structures under compression and shear loading. Composite Structures.

[30] Ullah I, Brandt M, Feih S (2016). Failure and energy absorption characteristics of advanced 3D truss core structures. Materials & Design.

[31] Wang, R. *et al*. Novel topological design of 3D Kagome structure for additive manufacturing. *Rapid Prototyping Journal*, 00–00, 10.1108/rpj-01-2017-0015 (2018).

[32] JinShui, Y. *Research on damping characteristic of composite lattice sandwich structures* Master thesis, Harbin Institute of Technology (2012).

[33] B. Wang, M. Y. Damping of honeycomb sandwich beams. *Journal of Materials Processing Technology* **105** (2000).

[34] Rao MD (2003). Recent applications of viscoelastic damping for noise control in automobiles and commercial airplanes. Journal of Sound and Vibration.

[35] Querin OM, Steven GP, Xie YM (2000). Evolutionary structural optimisation using an additive algorithm. Finite Elements in Analysis and Design.

[36] Canetta E, Duperray A, Leyrat A, Verdier C (2005). Measuring cell viscoelastic properties using a force-spectrometer: influence of protein-cytoplasm interactions. Biorheology.

